# The potential of virtual natural environments: a critical analysis of a VR-based mindfulness approach

**DOI:** 10.3389/fpsyg.2025.1637669

**Published:** 2025-07-23

**Authors:** Silvia Marocco, Valeria Vitale, Elena Grossi, Fabio Presaghi, Alessandra Talamo

**Affiliations:** Department of Social and Developmental Psychology, Sapienza University of Rome, Rome, Italy

**Keywords:** nature, virtual reality, mindfulness, emotions, environmental psychology

## Abstract

The relationship between environmental factors and mental health is widely acknowledged in the field of Environmental Psychology, with nature-based therapies emerging as a promising approach for mental health treatment. In particular, nature has been recognized as a valuable complementary resource for mindfulness practice. At the same time, Virtual Reality (VR) provides innovative solutions to the challenges posed by conventional mindfulness practices, reducing external distractions and fostering an immersive, present-focused experience. This study explores the effects of a nature VR-based and a conventional mindfulness intervention on the mindfulness state (decentering and curiosity) and emotions (positive and negative), with the aim of determining whether virtual nature can enhance mindfulness practice. The findings indicate that both conventional and VR-based mindfulness interventions effectively enhanced decentering; however, only the VR-based intervention significantly reduced negative emotions, suggesting that virtual natural environments may play a role in emotional regulation, as supported by previous research. On the other hand, no significant changes were observed in terms of curiosity or positive emotions across both intervention types. Overall, this study contributes to the understanding of how nature-based therapies and immersive technologies can support mental health treatment, providing a foundation for future research on the potential synergy between VR, mindfulness, and natural environments.

## 1 Introduction

The relationship between environmental factors and mental health is widely acknowledged, with nature-based therapies gaining recognition as a promising approach for mental health treatment. Studies in Environmental Psychology suggest that spending time in natural environments, such as green spaces and blue spaces, can significantly reduce stress (Schutte et al., 2017; Palanica et al., 2019; Schebella et al., 2020; Yu et al., 2018; Vitale et al., 2022; Grossi and Marocco, 2025; Marocco et al., 2025a,b), enhance physical activity, and promote social interaction—all of which contribute to improved mental well-being (Hartig et al., 2014; Roscoe et al., 2022).

Specifically, nature has been identified as a valuable complementary resource for mindfulness practice. Mindfulness plays a crucial role in promoting mental health and well-being by improving emotional awareness, enhancing emotional regulation, and reducing stress (Grossman et al., 2004; Vitale and Bonaiuto, 2024). Nevertheless, mindfulness requires conscious effort, which can be particularly challenging for beginners. Novices often expend significant cognitive resources to self-regulate their attention, making the practice more demanding and less accessible (Lymeus et al., 2017). Common difficulties include frustration, cognitive effort, environmental distractions, and adverse psychological outcomes, such as intrusive thoughts or distressing emotions (Anderson et al., 2019). To address these challenges, two promising solutions have emerged: exposure to nature and the use of immersive technologies.

On the one hand, leveraging the inherent qualities of natural environments or focusing on specific natural features can enhance mindfulness outcomes (Howell et al., 2011; Djernis et al., 2019). According to the Attention Restoration Theory (ART), indeed, exposure to natural settings reduces cognitive fatigue and improves attentional and restorative capacities, facilitating more effortless and focused meditation (Kaplan, 1980; Seabrook et al., 2020).

On the other hand, emerging technologies, particularly Virtual Reality (VR), offer innovative solutions to mitigate the challenges associated with mindfulness practice. Traditional mindfulness practices typically reduce sensory input by encouraging a fixed gaze and still posture, which can sometimes result in decreased alertness and the intrusion of unhelpful thoughts, especially for inexperienced practitioners (Navarro-Haro et al., 2017). In contrast, VR-based mindfulness engages attentional resources through visual and auditory stimuli, reducing the likelihood of external distractions and fostering an immersive, present-focused experience (Seabrook et al., 2020). In this perspective, VR does not aim to distract users but rather to facilitate active attention control, helping practitioners focus on the present moment (Navarro-Haro et al., 2017). However, it is crucial to recognize also the constraints that impact users’ willingness to adopt new technologies (Marocco et al., 2024c), such as the system usability, the user acceptance, and the domain suitability (Stickdorn and Schneider, 2012; Marocco et al., 2024a; Marocco et al., 2024b). Therefore, a human-centered design approach is crucial for effective VR interventions (Talamo et al., 2011; Talamo et al., 2021; Marocco and Talamo, 2022; Marocco et al., 2024a,b,c,d).

Research on VR-based mindfulness has shown mixed results. For instance, Navarro-Haro et al. (2017) found that VR-based mindfulness significantly increased the state of mindfulness and improved emotional states by reducing anxiety and sadness while enhancing relaxation. Similarly, Seabrook et al. (2020) showed that exposure to virtual natural environments significantly increased mindfulness and positive emotions, but did not result in notable changes in negative emotions or subjective arousal. Moreover, recent findings indicate that interoceptive awareness and decentering remain largely overlooked in the literature on VR-based mindfulness interventions (Arpaia et al., 2021).

Building on these considerations and addressing the existing research gap, the present study aims to enhance the understanding of how VR can support or hinder mindfulness practice. Specifically, the research seeks to determine the effect of a single mindfulness intervention, integrated with virtual natural environments, on the state of mindfulness (decentering and curiosity) and perceived emotions.

## 2 Research hypotheses

Based on the literature findings, we developed the following research hypotheses:

*H1*: *The effectiveness of VR-based Mindfulness sessions in enhancing the level of decentering (State of Mindfulness):*

*H1a: Pre-Post Effectiveness Analysis:* we hypothesize that overall the mindfulness interventions will increase the level of *decentering*, meaning the awareness of one’s experience with some distance and disidentification rather than being carried away by one’s thoughts and feelings (Teasdale et al., 2002).

*H1b: Comparison between Groups:* we hypothesize that the nature VR-based mindfulness intervention will lead to a greater increase of the level of *decentering*, compared to the conventional one.

*H2*: *The effectiveness of VR-based Mindfulness sessions in enhancing the level of curiosity (State of Mindfulness):*

*H2a: Pre-Post Effectiveness Analysis:* we hypothesize that overall the mindfulness interventions will increase the level of *curiosity*, meaning the awareness of the present moment experience (Teasdale et al., 2002).

*H2b: Comparison between Groups:* we hypothesize that nature VR-based mindfulness intervention will lead to a greater increase of the level of *curiosity*, compared to the conventional one.

*H3*: *The effectiveness of VR-based Mindfulness sessions in increasing Positive Emotions:*

*H3a: Pre-Post Effectiveness Analysis:* we hypothesize that overall the mindfulness interventions will increase positive emotions.

*H3b: Comparison between Groups:* we hypothesize that the nature VR-based mindfulness intervention will lead to a greater increase of positive emotions, compared to the conventional one.

*H4*: *The effectiveness of VR-based Mindfulness sessions in decreasing Negative Emotions:*

*H4a: Pre-Post Effectiveness Analysis:* we hypothesize that overall the mindfulness interventions will decrease negative emotions.

*H4b: Comparison between Groups:* we hypothesize that nature VR-based mindfulness intervention will lead to a greater decrease of negative emotions, compared to the conventional one.

## 3 Materials and methods

### 3.1 Research design

The present study adopts a mixed-methods research design with the aim of investigating the impact of virtual natural scenarios (between-subjects factor: experimental condition with or without virtual natural scenarios) on the state of mindfulness and emotions’ elicitation (within-subjects factor: pre- and post-measurements of the experimental session).

### 3.2 Sample description

The study recruited Italian young adults aged 18–40, aiming for a balanced gender distribution. A sample size calculation, performed via *a priori* power analysis using GPower (version 3.1.9.6.), indicated that a minimum of 34 participants were required to achieve 80% power, a medium effect size (*f* = 0.25), and a significance level of *α* ≤ 0.05 for the main analyses of interest. Based on this, the study targeted a total of 50 participants (25 per experimental group). The recruitment process involved an online questionnaire distributed via snowball sampling techniques. A total of 90 responses were collected, comprising 61 women and 29 men (M age = 25.12, SD = 3.46, range: 20–43 years). For the screening phase, participants with prior mindfulness experience were excluded from the sample. This was important to control for any pre-existing effects of mindfulness on their emotional state or mindfulness levels, ensuring that the study accurately assessed the impact of the intervention on naive participants. Specifically, 11 individuals were excluded due to prior engagement in mindfulness practices. The final sample consisted of 49 participants who voluntarily chose to take part in the study, including 31 women (63.3%) and 18 men (36.7%). The average age of participants was 24.86 years (SD = 3.01), predominantly student-based sample (79.6%), holding a bachelor degree (65.3%).

### 3.3 Procedure

Participants first completed a recruitment questionnaire, which gathered socio-demographic information (gender, age, education, place of birth, occupation), questions about their prior experience with VR and mindfulness, and their connection to nature. Those who met the inclusion criteria were contacted via email to schedule the experimental session, providing them with basic procedural information and instructions.

The study was conducted in the IDEaCT Social Lab at the Department of Social and Developmental Psychology, Sapienza University of Rome.

Upon arrival, participants read and signed an informed consent form explaining the study’s objectives, procedures, data treatment, and potential short-term effects of VR use. They were then fitted with the Polar H10 heart rate sensor, linked to the Elite HRV application, to measure baseline heart rate and inter-beat interval data for 2 min.

The Oculus Quest 2, a standalone VR system with an HMD and two manual controllers, was provided. Participants also wore over-ear headphones to hear environmental sounds (e.g., wind, birds, waves). A video recording of the session began, which included the lab space and mirrored VR footage to monitor participants’ behaviors.

Participants were instructed to select and explore three virtual natural environments— a tropical island, a meadow, and a forest. The order of these scenarios was randomized. After each 2-min exploration, they removed the headset to complete a questionnaire on a computer, assessing their subjective emotional experience and the explored environment.

Once all three environments were explored, participants completed a questionnaire on their current emotional state (PANAS) and indicated their preferred scenario for the subsequent mindfulness session, which would use VR in the experimental condition.

The mindfulness session involved a 23-min audio track, combining a guiding voice and music at 432 Hz to stimulate brain alpha waves and beta-endorphins. The guided mindfulness practice used in this study was taken from the *Mindfulness-Based Stress Reduction (MBSR)* program developed by Woods and Rockman (2021), in its Italian adaptation (Woods and Rockman, 2022). The audio track features a professional instructor guiding participants through a structured mindfulness meditation consistent with MBSR principles. The guidance follows a gentle, non-directive style, with a calm and steady tone that encourages present-moment awareness and acceptance. The practice includes key themes typical of MBSR, such as focused attention on the breath, non-judgmental awareness of bodily sensations, and the invitation to gently return attention when the mind wanders. A single audio track was created for both experimental conditions, with the only difference being that in the conventional mindfulness track, participants were instructed to close their eyes, whereas this detail was omitted in the track for VR-based mindfulness. Participants listened to the track while seated in a chair at the center of the room. In the VR-based mindfulness condition, participants wore the headset again to view their preferred scenario.

Following the mindfulness experience, participants completed a final questionnaire assessing their emotional state (PANAS) and mindfulness state. They were also asked about their subjective experience with the virtual scenarios, the usability of the VR system, any symptoms related to headset use, and their sense of immersion and presence in the virtual environment.

### 3.4 Virtual scenarios

**Figure 1:**
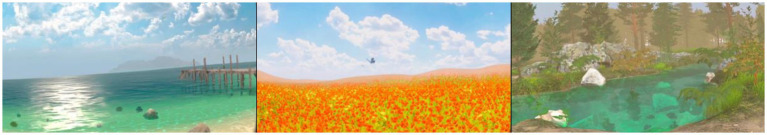
Screenshots of the natural scenarios within virtual reality (in order: “tropical island,” “meadow,” “forest”).

The virtual natural scenarios (Figure 1) were developed following the eight components of the Biophilic Effect (Salingaros, 2015), which include sunlight, color, gravity, fractal elements, curves, details, water, and life. These elements are known to promote relaxation and stress recovery, offering a healing effect on the human body. Specifically, the three natural scenarios analyzed in this study represent a tropical island, a meadow, and a forest.

### 3.5 Measures

In the recruitment survey, participants completed a series of questions assessing socio-demographic indicators, including gender, age, highest level of educational achievement, country of birth, and employment status. They were also asked about prior experience with mindfulness and VR, dispositional mindfulness, and their connection with nature.

Dispositional mindfulness, serving as the pre-test measurement, was measured using the Italian version of the *Toronto Mindfulness Trait Scale* (TMTS; Davis et al., 2009), which includes 13 items divided into two dimensions. The first one is curiosity which reflects an individual’s disposition to explore and understand their experience. In particular, it refers to the interest in every aspect of inner experience, including thoughts, emotions and bodily sensations, observed with an attitude of openness and exploration (Lau et al., 2006) even when they are unpleasant. Examples of items are: “*I am curious about what I might learn about myself by taking notice of how I react to certain thoughts, feelings or sensations*” and “*I remain curious about the nature of each experience as it arises*.”

The second one is decentering, indicating the tendency to observe thoughts and emotions without becoming absorbed by them. Specifically, it is defined as the ability to observe thoughts and emotions as temporary mental events rather than as accurate reflections of the self or reality (Lau et al., 2006), a capacity that plays a particularly important role in emotional regulation. Examples of items are: “*I am more invested in just watching my experiences as they arise, than in figuring out what they could mean*” and “*I am aware of my thoughts and feelings without overidentifying with them.”*

The *Toronto Mindfulness State Scale* (TMS; Lau et al., 2006) was adopted to assess - as the post-test measurement - the dimensions of curiosity and decentering through 13 items, with participants rating their experiences during the meditation on a 5-point scale ranging from 0 (“not at all”) to 4 (“very much”).

The Italian short version of the *Positive and Negative Affect Schedule* (PANAS; Watson et al., 1988; Terracciano et al., 2003) was used to assess participants’ emotional states pre- and post- intervention. The Positive Affect scale assesses the degree of pleasurable engagement with the environment, characterized by emotions such as enthusiasm, excitement, energy, and determination. Conversely, the Negative Affect scale captures a general dimension of subjective distress and unpleasurable engagement, encompassing a broad spectrum of aversive emotional states, including fear, nervousness, guilt, and shame (Terracciano et al., 2003). This scale evaluates positive and negative emotions through self-reported agreement with items on a 5-point Likert scale.

## 4 Results

Analytic strategy plan is detailed in Supplementary material S1. Additionally, preliminary analyses were conducted on dispositional mindfulness and baseline emotions to assess groups comparability (see Supplementary material S2).

### 4.1 The effectiveness of VR-based mindfulness sessions in enhancing the level of decentering (H1)

Table 1 shows descriptive statistics of the variables of interest across groups.

**Table 1:** Descriptive statistics with mean and standard deviation (in parentheses) for the variables of interest at pre- and post-intervention across groups.

Variable	Group	Mean (SD)	
		Pre-test	Post-test
Mindfulness—Decentering	Mindfulness-only	13.78 (3.05)	19.13 (3.15)
	Mindfulness + VR	13.96 (3.15)	18.30 (3.71)
Mindfulness—Curiosity	Mindfulness-only	19.39 (3.91)	19.44 (3.34)
	Mindfulness + VR	19.35 (2.77)	19.23 (4.01)
Emotions—Positive	Mindfulness-only	3.35 (0.68)	3.31 (0.80)
	Mindfulness + VR	3.29 (0.73)	3.24 (0.77)
Emotions—Negative	Mindfulness-only	1.38 (0.55)	1.13 (0.35)
	Mindfulness + VR	1.40 (0.58)	1.06 (0.17)

**Figure 2:**
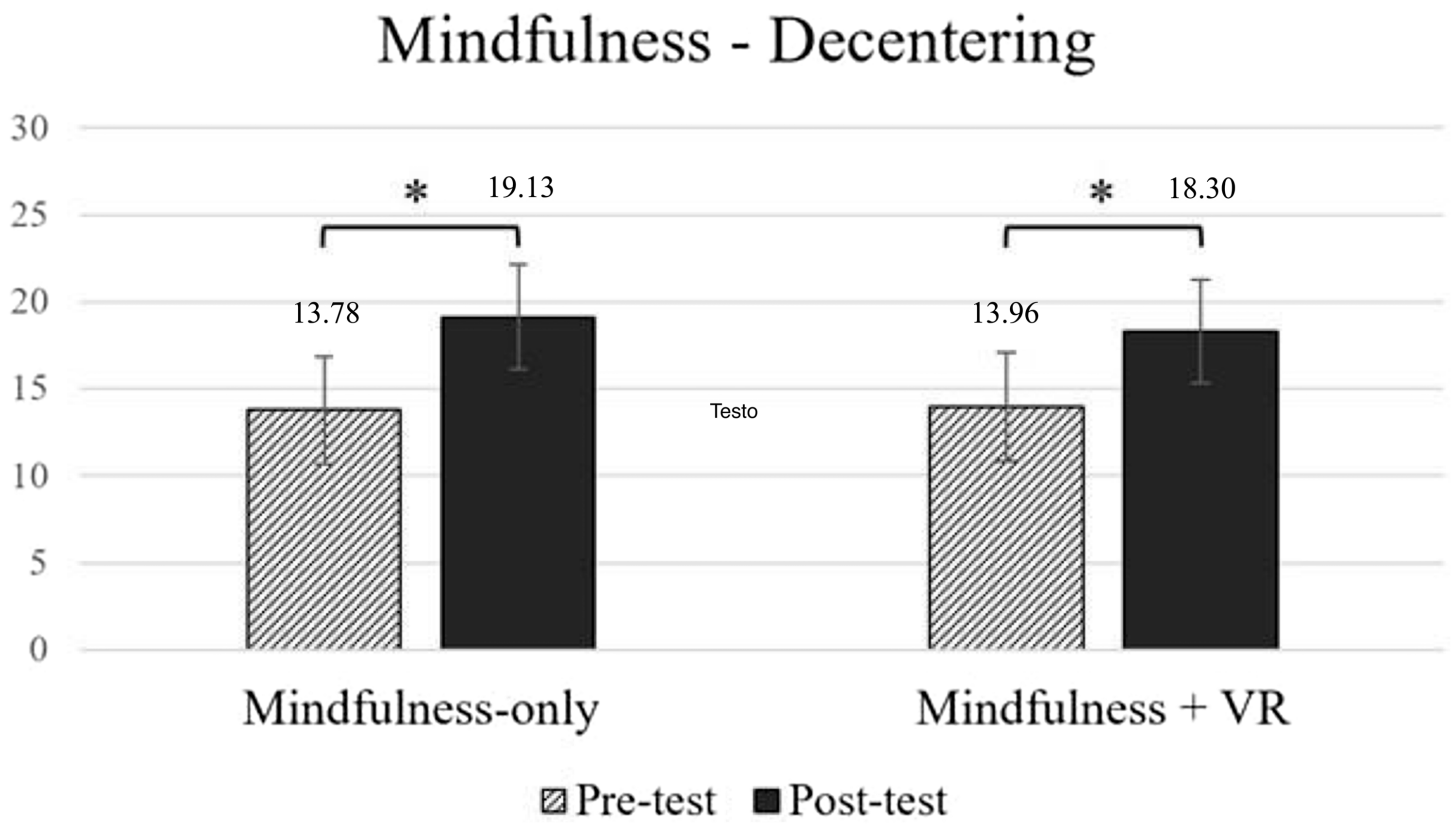
Changes in decentering before and after intervention across groups. * indicates a significant difference in comparison with variable scores.

Concerning decentering (Figure 2), a significant main effect of time was observed, *F* (1, 47) = 77.102, *p* < 0.001, *η^2^_p_* = 0.621, indicating overall higher scores at the state level post-intervention (*M* = 13.88, SD = 3.07) than at the trait level pre-intervention (*M* = 18.69, SD = 3.45). However, no significant main effect of the experimental group was found, *F* (1, 47) = 0.178, *p* = 0.675, *η^2^_p_* = 0.004. Additionally, the interaction between time and experimental group was not significant, *F* (1, 47) = 0.823, *p* = 0.369, *η^2^_p_* = 0.017.

Post-hoc comparisons showed that the increase of decentering level in the post-intervention was significant for both the VR-based mindfulness group, *t* (47) = −5.746, *p_tukey_* < 0.001, and the conventional group, *t* (47) = −6.650, *p_tukey_* < 0.001. For a comprehensive overview of the analysis, please see the Supplementary material S3.

This result indicates that the increase in decentering after the intervention was observed across all participants, regardless of whether they engaged in mindfulness alone or mindfulness combined with nature VR, suggesting the intervention type did not differentially impact decentering.

### 4.2 The effectiveness of VR-based mindfulness sessions in enhancing the level of curiosity (H2)

Regarding curiosity (Supplementary Figure S1), no significant main effects of time, *F* (1, 47) = 0.004, *p* = 0.951, *η^2^_p_* = 0.000, nor of the experimental group factor were observed, *F* (1, 47) = 0.023, *p* = 0.881, *η^2^_p_* = 0.000. Also, results did not show a significant interaction between time and experimental group, *F* (1, 47) = 0.019, *p* = 0.892, *η^2^_p_* = 0.000. As reported, the curiosity scores tend to vary very slightly after the intervention (*M* = 19.33, SD = 3.68) compared to the pre-test (*M* = 19.37, SD = 3.32): it seems that the curiosity dimension is not affected by the Mindfulness intervention both when combined or not with VR. Complete analysis details are provided in the Supplementary material S4.

### 4.3 The effectiveness of VR-based mindfulness sessions in increasing positive emotions (H3)

Regarding positive emotions (Supplementary Figure S2), the main effect of time was not significant, *F* (1, 47) = 0.144, *p* = 0.706, *η^2^_p_* = 0.003, so there were no relevant differences between positive emotions detected during the pre (*M* = 3.31, SD = 0.70) and the post test (*M* = 3.28, SD = 0.79). As the graph shows, the main effect of the experimental group, *F* (1, 47) = 0.001, *p* = 0.982, *η^2^_p_* = 0.000 and the interaction effect of time and experimental group, were not significant too, *F* (1, 47) = 0.486, *p* = 0.489, *η^2^_p_* = 0.010. Further details of the analysis can be found in the Supplementary material S5.

Overall, it seems that the proposed interventions have no significant effect in enhancing positive emotions, contrasting our initial hypothesis.

### 4.4 The effectiveness of VR-based mindfulness sessions in increasing negative emotions (H4)

**Figure 3:**
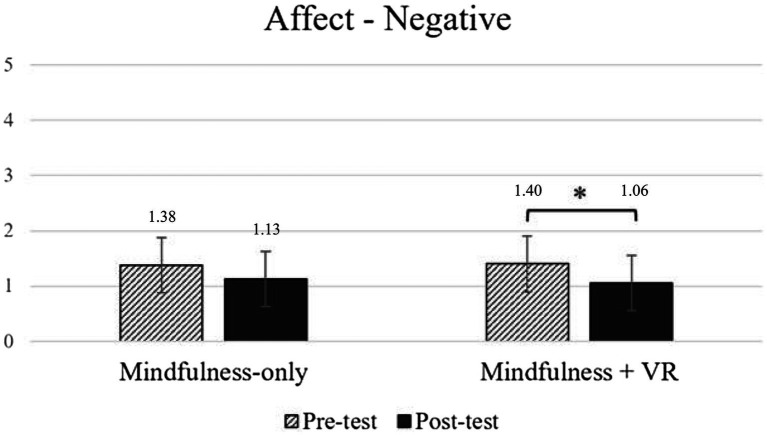
Changes in negative emotions before and after intervention across groups. * indicates a significant difference in comparison with variable scores.

Concerning negative emotions (Figure 3), according to the analysis, there is a significant main effect of time, *F* (1, 47) = 15.732, *p* < 0.001, *η^2^_p_* = 0.251: the negative emotions perception decrease from the pre-test (*M* = 1.40, SD = 0.57) to the post-test (*M* = 1.10, SD = 0.27), as expected. In contrast, both the main effect of the experimental group, *F* (1, 47) = 0.030, *p* = 0.864, *η^2^_p_* = 0.001, and the interaction effect of time and experimental group were not significant, *F* (1, 47) = 0.336, *p* = 0.565, *η^2^_p_* = 0.007.

However, post-hoc comparisons revealed that the reduction in negative emotions from pre- to post-test was significant only for participants in the experimental group who underwent VR-based mindfulness, *t* (47) = 3.318, *p_tukey_* = 0.009. In contrast, the difference between pre- and post-test scores was not significant in the group that received conventional mindfulness, *t* (47) = 2.325, *p_tukey_* = 0.107.

Refer to the Supplementary material S6 for a full description of the analysis.

Thus, it appears that VR-based mindfulness significantly reduced negative emotions, whereas conventional mindfulness did not show a comparable effect, suggesting that experiencing virtual nature may enhance the effectiveness of mindfulness practices in reducing negative emotions.

## 5 Discussion

The present study adopts a mixed-methods research design with the aim of investigating the impact of nature VR-based mindfulness session on the state of mindfulness and emotions’ elicitation.

The results of this study partially confirmed H1. Specifically, H1a was supported. Indeed, an increase in the state of mindfulness in terms of decentering after the intervention was observed. This finding suggests that overall the interventions were effective in enabling participants to adopt a more “decentered” perspective on their thoughts and emotions. On the other hand, H1b was not supported. In fact, significant differences in mindfulness scores of decentering were found across both groups, meaning that the VR-based mindfulness intervention did not produce significantly higher effects compared to the conventional mindfulness intervention.

Also H2a was not verified, as no significant changes in curiosity scores were observed before and after interventions and no significant differences were found across groups.

Similarly, H3 was not supported, as there was no significant pre-post intervention difference in positive emotions, nor were there significant differences between the two groups.

Conversely, H4 was confirmed. Specifically, H4a was supported, since a significant decrease in negative emotions were observed post intervention. Moreover, H4b was verified since the reduction of negative emotions was significant only in the VR-based mindfulness group, whereas no significant differences were found in the conventional mindfulness group.

Overall, although no significant pre-post changes nor differences across groups were observed in terms of curiosity and positive emotions, the study results appear to be aligned with previous literature. Indeed, the VR-based mindfulness intervention was able to reduce negative emotions, similarly to what has been reported by Navarro-Haro et al. (2017). This suggests that virtual environments may contribute to emotional regulation, aligning with findings from previous studies (Lau et al., 2006; Choe et al., 2020b). Additionally, the well-established restorative effects of natural settings (Kaplan, 1980; Ulrich, 1983) likely support emotional regulation by alleviating negative emotions. Several studies (Djernis et al., 2019; Choe et al., 2020a) have explored this by conducting mindfulness sessions in natural physical environments, reporting that mindfulness states tend to improve more significantly in natural settings compared to non-natural ones.

Finally, findings suggest that both conventional and VR-based mindfulness interventions effectively enhanced decentering. While the effects of conventional mindfulness on decentering are well-established in the literature (Shoham et al., 2017), the novelty of this study lies in the finding that VR-based mindfulness may also facilitate a decentering experience. The strong sense of presence typically evoked during exposure to virtual scenarios (Lønne et al., 2023) may have contributed to a more detached perspective on personal thoughts, thereby supporting the experience of decentering.

### 5.1 Limitations and future research

The present study showed some promising results; however, some limitations should be considered. A possible explanation for the discrepancy between some observed results and our expectations, particularly regarding the expected added value of nature in VR, could be linked to some issues due to the design of the intervention, specifically the duration of the VR-based meditation session and the modality of experiencing virtual natural scenarios. Observing a virtual scene for 23 consecutive minutes without the option for interaction or exploration—since the meditation was conducted in a seated position—could have been a limiting factor. Mindfulness practice in natural environments - whether real or simulated - relies on attentional anchors and sensory stimuli to help practitioners stay present and engaged (Brown and Ryan, 2003). While the virtual environments in this study were visually and auditorily rich, it is possible that after a certain period, they ceased to be stimulating, failing to maintain participants’ attention. Additionally, several participants in the VR-based group expressed that they would have preferred to close their eyes during the mindfulness practice. This may have led them to perceive the VR experience as a barrier, preventing complete immersion in the practice. Future studies could further explore these aspects to determine the most effective way to structure the experience of mindfulness sessions in natural virtual environments. Additionally, research should investigate whether—and to what extent—specific characteristics of the natural environment influence the effectiveness of mindfulness practice.

Moreover, while participants with prior mindfulness experience were excluded to control for pre-existing differences in familiarity with the practice, this decision limits the generalizability of the findings to novice practitioners. Evidence suggests that experienced meditators may engage differently with mindfulness-based interventions, both physiologically and neurologically. For example, experienced practitioners exhibit reduced heart rate variability, reflecting enhanced autonomic self-regulation (Peressutti et al., 2012), and show altered resting-state connectivity in brain regions associated with self-referential processing and cognitive control, such as the posterior cingulate cortex and dorsolateral prefrontal cortex (Kral et al., 2018). Supporting this, Falcone and Jerram’s (2018) meta-analysis found distinct patterns of brain activation between novice and experienced meditators, indicating that mindfulness is a learned skill characterized by progressive neuroplastic changes—such as shifts from insula activation in novices to basal ganglia involvement in experts—reflecting increasing habitual engagement with the practice. To deepen understanding and optimize intervention efficacy, future research should systematically investigate how varying levels of prior mindfulness experience influence behavioral outcomes as well as underlying neural and physiological mechanisms. This will facilitate the development of more tailored and effective mindfulness-based interventions, including combined VR nature-based mindfulness interventions across diverse populations.

## 6 Conclusion

This study investigated the impact of nature VR-based mindfulness on mindfulness and emotional responses. The results indicate that both conventional and VR-based mindfulness interventions successfully promoted decentering, but only the VR-based intervention led to a reduction in negative emotions. However, no significant changes were observed in curiosity or positive emotions before and after the intervention, nor were there differences between the groups.

Given that this study assessed the immediate effects of a single exposure to virtual nature during mindfulness practice, further research is needed to explore the optimal duration of exposure and to evaluate the long-term impact of VR-supported mindfulness practice. Additionally, future studies should consider integrating interactive VR environments that allow for controller-based locomotion or other forms of exploration, even in a seated position, to enhance engagement and sense of presence.

Overall, this study contributes to the understanding of how nature-based therapies and the use of immersive technologies can support mental health treatment and lays the groundwork for future research on the potential synergy between VR and mindfulness-based interventions.

## Data Availability

The raw data supporting the conclusions of this article will be made available by the authors, without undue reservation.
