# Supplementary material for: The potential of virtual natural environments: a critical analysis of a VR-based mindfulness approach
Source: Front Psychol. 2025 Jul 23;16:1637669. doi: 10.3389/fpsyg.2025.1637669 (PMC12325303; doi:10.3389/fpsyg.2025.1637669)
Supplement: Supplementary file 1 [file Table_1.DOCX]

**Supplementary materials**

**The potential of Virtual Natural Environments:**

**a Critical Analysis of a VR-based Mindfulness Approach**

**S1. *Analytic strategies***

Descriptive analyses were performed to examine participants' demographic characteristics and the variables of interest.

To ensure equivalence between the two participant groups—one experiencing a VR-based mindfulness session (experimental group) and the other a conventional mindfulness session (control group) preliminary analyses were conducted. A series of ANOVAs were adopted to examine baseline similarity in emotional states (PANAS), and Dispositional Mindfulness (TMTS), with group assignment as a between-subjects factor.

Additionally, data has been used to verify our research hypotheses. Specifically, a series of mixed design ANOVA with a within-subjects factor (pre- and post-measurements of the experimental session), and a between-subjects factor (experimental condition with or without virtual natural scenarios), was performed to investigate changes in the state of Mindfulness scores and PANAS measures before and after the Mindfulness sessions.

These analyses investigated whether overall the interventions were able to enhance decentering and curiosity - components of the state of Mindfulness (H1a, H2a) - as well as promoting positive emotions (H3a) and reduce negative emotions (H4a). Furthermore, we aimed to determine whether exposure to nature VR-based mindfulness was more effective (H1b, H2b, H3b, H4b).

Specifically, we were interested in the following effects: the main effect of time (changes pre-post); the main effect of the group (differences between experimental and control groups); the interaction effect of time and group (changes pre-post as a function of the experimental condition).

Moreover, additional analyses were performed on ECG physiological parameters (SNS and PNS) in terms of baseline similarity and differences across time and group. For brevity, these analyses are not reported in this article.

**S2. *Preliminary analysis***

***Pre-test PANAS (positive and negative) across experimental groups***

An univariate ANOVA with a between-subjects factor representing the two groups was conducted to verify the groups equivalence in terms of positive and negative affect (PANAS). No significant differences emerge both for negative affect values, *F*(1,47) = 0.023, *p* = 0.879, *η²_p_* =  0.000, and positive affect values, *F*(1,47) = 0.098, *p* = 0.755, *η²_p_* =  0.002. According to these results, the two groups appear to be equivalent in terms of positive and negative affect in the pre-test phase.

***Dispositional Mindfulness (TMTS) across experimental groups***

An ANOVA was conducted to examine differences in the scores of the two dimensions of dispositional mindfulness (TMTS) among participants in the two experimental groups. These preliminary analyses aimed to confirm that all participants had similar levels of dispositional mindfulness, regardless of their experimental group. No significant differences were observed in the *DeCentering* dimension, *F*(1, 47) = 0.041, *p* = 0.841, *η²_p_*= 0.001, or the *Curiosity* dimension, *F*(1, 47) = 0.002, *p* = 0.963, *η²_p_* = 0.000, of dispositional mindfulness between the experimental conditions. Table 1 presents the means and standard deviations for the two dimensions of dispositional mindfulness across both experimental groups.

**S3. *Full analysis of mindfulness – decentering***

| Within Subjects Effects | | | | | | | |
| --- | --- | --- | --- | --- | --- | --- | --- |
|  | ***Sum of Squares*** | ***df*** | ***Mean Square*** | ***F*** | ***p*** | ***η²*** | ***η²_p_*** |
| Time | 573.429 | 1 | 573.429 | 77.102 | < .001 | 0.359 | 0.621 |
| Time ✻ Group | 6.122 | 1 | 6.122 | 0.823 | 0.369 | 0.004 | 0.017 |
| Residual | 349.551 | 47 | 7.437 |  |  |  |  |

| Between Subjects Effects | | | | | | | |
| --- | --- | --- | --- | --- | --- | --- | --- |
|  | ***Sum of Squares*** | ***df*** | ***Mean Square*** | ***F*** | ***p*** | ***η²*** | ***η²_p_*** |
| Group | 2.529 | 1 | 2.529 | 0.178 | 0.675 | 0.002 | 0.004 |
| Residual | 667.471 | 47 | 14.202 |  |  |  |  |

| Post Hoc Comparisons – Time | | | | | | | |
| --- | --- | --- | --- | --- | --- | --- | --- |
| **Comparison** | | |  | | | | |
| **Time** |  | **Time** | ***Mean Difference*** | ***SE*** | ***df*** | ***t*** | ***p_tukey_*** |
| Trait | - | State | -4.847 | 0.552 | 47 | -8.781 | < .001 |

| Post Hoc Comparisons – Group | | | | | | | |
| --- | --- | --- | --- | --- | --- | --- | --- |
| **Comparison** | | |  | | | | |
| **Group** |  | **Group** | ***Mean Difference*** | ***SE*** | ***df*** | ***t*** | ***p_tukey_*** |
| Mindfulness + VR | - | Mindfulness-only | -0.322 | 0.763 | 47 | -0.422 | 0.675 |

| Post Hoc Comparisons – Time ✻ Group | | | | | | | | | | |
| --- | --- | --- | --- | --- | --- | --- | --- | --- | --- | --- |
| **Comparison** | | | | | |  | | | | |
| **Time** | **Group** |  | **Time** | **Group** | ***Mean Difference*** | | ***SE*** | ***df*** | ***t*** | ***p_tukey_*** |
| Trait | Mindfulness + VR | - | Trait | Mindfulness-only | 0.179 | | 0.889 | 47 | 0.201 | 0.997 |
|  |  | - | State | Mindfulness + VR | -4.346 | | 0.756 | 47 | -5.746 | < .001 |
|  |  | - | State | Mindfulness-only | -5.169 | | 0.945 | 47 | -5.471 | < .001 |
|  | Mindfulness-only | - | State | Mindfulness + VR | -4.525 | | 0.938 | 47 | -4.822 | < .001 |
|  |  | - | State | Mindfulness-only | -5.348 | | 0.804 | 47 | -6.650 | < .001 |
| State | Mindfulness + VR | - | State | Mindfulness-only | -0.823 | | 0.992 | 47 | -0.830 | 0.840 |

**S4. *Full analysis of mindfulness – curiosity***

| Within Subjects Effects | | | | | | | |
| --- | --- | --- | --- | --- | --- | --- | --- |
|  | ***Sum of Squares*** | ***df*** | ***Mean Square*** | ***F*** | ***p*** | ***η²*** | ***η²_p_*** |
| Time | 0.032 | 1 | 0.032 | 0.004 | 0.951 | 0.000 | 0.000 |
| Time ✻ Group | 0.154 | 1 | 0.154 | 0.019 | 0.892 | 0.000 | 0.000 |
| Residual | 386.805 | 47 | 8.230 |  |  |  |  |

| Between Subjects Effects | | | | | | | |
| --- | --- | --- | --- | --- | --- | --- | --- |
|  | ***Sum of Squares*** | ***df*** | ***Mean Square*** | ***F*** | ***p*** | ***η²*** | ***η²_p_*** |
| Group | 0.379 | 1 | 0.379 | 0.023 | 0.881 | 0.000 | 0.000 |
| Residual | 788.825 | 47 | 16.784 |  |  |  |  |

| Post Hoc Comparisons – Time | | | | | | | |
| --- | --- | --- | --- | --- | --- | --- | --- |
| **Comparison** | | |  | | | | |
| **Time** |  | **Time** | ***Mean Difference*** | ***SE*** | ***df*** | ***t*** | ***p_tukey_*** |
| Trait | - | State | 0.036 | 0.581 | 47 | 0.062 | 0.951 |

| Post Hoc Comparisons – Group | | | | | | | |
| --- | --- | --- | --- | --- | --- | --- | --- |
| **Comparison** | | |  | | | | |
| **Group** |  | **Group** | ***Mean Difference*** | ***SE*** | ***df*** | ***t*** | ***p_tukey_*** |
| Mindfulness + VR | - | Mindfulness-only | -0.125 | 0.829 | 47 | -0.150 | 0.881 |

| Post Hoc Comparisons – Time ✻ Group | | | | | | | | | | |
| --- | --- | --- | --- | --- | --- | --- | --- | --- | --- | --- |
| **Comparison** | | | | | |  | | | | |
| **Time** | **Group** |  | **Time** | **Group** | ***Mean Difference*** | | ***SE*** | ***df*** | ***t*** | ***p_tukey_*** |
| Trait | Mindfulness + VR | - | Trait | Mindfulness-only | -0.045 | | 0.959 | 47 | -0.047 | 1.000 |
|  |  | - | State | Mindfulness + VR | 0.115 | | 0.796 | 47 | 0.145 | 0.999 |
|  |  | - | State | Mindfulness-only | -0.089 | | 1.016 | 47 | -0.087 | 1.000 |
|  | Mindfulness-only | - | State | Mindfulness + VR | 0.161 | | 1.009 | 47 | 0.159 | 0.999 |
|  |  | - | State | Mindfulness-only | -0.043 | | 0.846 | 47 | -0.051 | 1.000 |
| State | Mindfulness + VR | - | State | Mindfulness-only | -0.204 | | 1.063 | 47 | -0.192 | 0.997 |

**Fig. S1.** *Changes in Curiosity before and after intervention across groups*

**
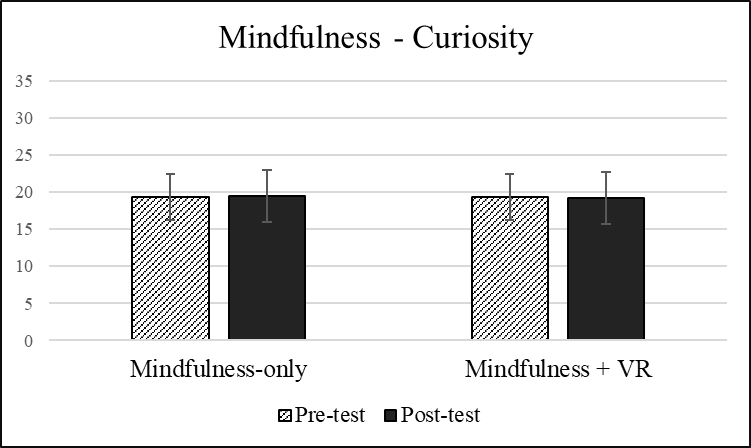
**

**S5. *Full analysis of PANAS – positive affect***

| Within Subjects Effects | | | | | | | |
| --- | --- | --- | --- | --- | --- | --- | --- |
|  | ***Sum of Squares*** | ***df*** | ***Mean Square*** | ***F*** | ***p*** | ***η²*** | ***η²_p_*** |
| Time | 0.033 | 1 | 0.033 | 0.144 | 0.706 | 0.001 | 0.003 |
| Time ✻ Group | 0.111 | 1 | 0.111 | 0.486 | 0.489 | 0.002 | 0.010 |
| Residual | 10.782 | 47 | 0.229 |  |  |  |  |

| Between Subjects Effects | | | | | | | |
| --- | --- | --- | --- | --- | --- | --- | --- |
|  | ***Sum of Squares*** | ***df*** | ***Mean Square*** | ***F*** | ***p*** | ***η²*** | ***η²_p_*** |
| Group | 0.000 | 1 | 0.000 | 0.001 | 0.982 | 0.000 | 0.000 |
| Residual | 42.139 | 47 | 0.897 |  |  |  |  |

| Post Hoc Comparisons – Time | | | | | | | |
| --- | --- | --- | --- | --- | --- | --- | --- |
| **Comparison** | | |  | | | | |
| **Time** |  | **Time** | ***Mean Difference*** | ***SE*** | ***df*** | ***t*** | ***p_tukey_*** |
| Pre | - | Post | 0.037 | 0.097 | 47 | 0.379 | 0.706 |

| Post Hoc Comparisons – Group | | | | | | | |
| --- | --- | --- | --- | --- | --- | --- | --- |
| **Comparison** | | |  | | | | |
| **Group** |  | **Group** | ***Mean Difference*** | ***SE*** | ***df*** | ***t*** | ***p_tukey_*** |
| Mindfulness + VR | - | Mindfulness-only | 0.004 | 0.192 | 47 | 0.023 | 0.982 |

| Post Hoc Comparisons - Time ✻ Group | | | | | | | | | |
| --- | --- | --- | --- | --- | --- | --- | --- | --- | --- |
| **Comparison** | | | | |  | | | | |
| **Time** | **Group** |  | **Time** | **Group** | ***Mean Difference*** | ***SE*** | ***df*** | ***t*** | ***p_tukey_*** |
| Pre | Mindfulness + VR | - | Pre | Mindfulness-only | -0.063 | 0.202 | 47 | -0.314 | 0.989 |
|  |  | - | Post | Mindfulness + VR | -0.031 | 0.133 | 47 | -0.232 | 0.996 |
|  |  | - | Post | Mindfulness-only | 0.041 | 0.216 | 47 | 0.191 | 0.998 |
|  | Mindfulness-only | - | Post | Mindfulness + VR | 0.032 | 0.214 | 47 | 0.152 | 0.999 |
|  |  | - | Post | Mindfulness-only | 0.104 | 0.141 | 47 | 0.739 | 0.881 |
| Post | Mindfulness + VR | - | Post | Mindfulness-only | 0.072 | 0.227 | 47 | 0.316 | 0.989 |

**Fig. S2.** *Changes in Positive Emotions before and after intervention across groups*

**
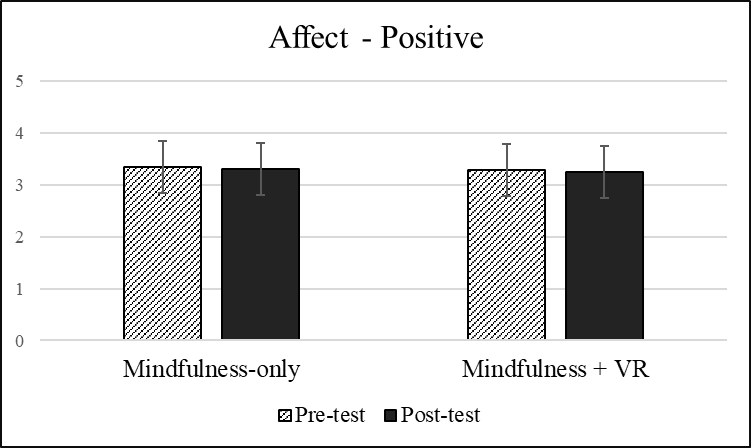
**

**S6. *Full analysis of PANAS – negative affect***

| Within Subjects Effects | | | | | | | |
| --- | --- | --- | --- | --- | --- | --- | --- |
|  | ***Sum of Squares*** | ***df*** | ***Mean Square*** | ***F*** | ***p*** | ***η²*** | ***η²_p_*** |
| Time | 2.129 | 1 | 2.129 | 15.732 | < .001 | 0.101 | 0.251 |
| Time ✻ Group | 0.045 | 1 | 0.045 | 0.336 | 0.565 | 0.002 | 0.007 |
| Residual | 6.359 | 47 | 0.135 |  |  |  |  |

| Between Subjects Effects | | | | | | | |
| --- | --- | --- | --- | --- | --- | --- | --- |
|  | ***Sum of Squares*** | ***df*** | ***Mean Square*** | ***F*** | ***p*** | ***η²*** | ***η²_p_*** |
| Group | 0.008 | 1 | 0.008 | 0.030 | 0.864 | 0.000 | 0.001 |
| Residual | 12.596 | 47 | 0.268 |  |  |  |  |

| Post Hoc Comparisons – Time | | | | | | | |
| --- | --- | --- | --- | --- | --- | --- | --- |
| **Comparison** | | |  | | | | |
| **Time** |  | **Time** | ***Mean Difference*** | ***SE*** | ***df*** | ***t*** | ***p_tukey_*** |
| Pre | - | Post | 0.295 | 0.074 | 47 | 3.966 | <.001 |

| Post Hoc Comparisons – Group | | | | | | | |
| --- | --- | --- | --- | --- | --- | --- | --- |
| **Comparison** | | |  | | | | |
| **Group** |  | **Group** | ***Mean Difference*** | ***SE*** | ***df*** | ***t*** | ***p_tukey_*** |
| Mindfulness + VR | - | Mindfulness-only | -0.018 | 0.105 | 47 | -0.172 | 0.864 |

| Post Hoc Comparisons - Time ✻ Group | | | | | | | | | |
| --- | --- | --- | --- | --- | --- | --- | --- | --- | --- |
| **Comparison** | | | | |  | | | | |
| **Time** | **Group** |  | **Time** | **Group** | ***Mean Difference*** | ***SE*** | ***df*** | ***t*** | ***p_tukey_*** |
| Pre | Mindfulness + VR | - | Pre | Mindfulness-only | 0.025 | 0.164 | 47 | 0.153 | 0.999 |
|  |  | - | Post | Mindfulness + VR | 0.338 | 0.102 | 47 | 3.318 | 0.009 |
|  |  | - | Post | Mindfulness-only | 0.277 | 0.126 | 47 | 2.199 | 0.138 |
|  | Mindfulness-only | - | Post | Mindfulness + VR | 0.313 | 0.131 | 47 | 2.393 | 0.092 |
|  |  | - | Post | Mindfulness-only | 0.252 | 0.108 | 47 | 2.325 | 0.107 |
| Post | Mindfulness + VR | - | Post | Mindfulness-only | -0.061 | 0.079 | 47 | -0.776 | 0.865 |
